# A hybrid strategy using an ambulance and a helicopter to convey thrombectomy candidates to definite care: a prospective observational study

**DOI:** 10.1186/s12873-024-00931-0

**Published:** 2024-01-25

**Authors:** Pauli Vuorinen, Piritta Setälä, Jyrki Ollikainen, Sanna Hoppu

**Affiliations:** 1 Emergency Medical Services, Centre for Prehospital Emergency Care, Pirkanmaa wellbeing services county, Tampere, Finland; 2 Faculty of Medicine and Health Technology, University of Tampere, Tampere, Finland; 3 Department of Neurosciences and Rehabilitation, Tampere University Hospital, Tampere, Finland; 4 Emergency Medical Services, Tampere University Hospital, FI-33521 Tampere, PO Box 2000, Finland

**Keywords:** Helicopter emergency medicine service, Large vessel occlusion stroke, Mechanical thrombectomy

## Abstract

**Background:**

Mechanical thrombectomy is the treatment of choice for large vessel occlusion strokes done only in comprehensive stroke centres (CSC). We investigated whether the transportation time of thrombectomy candidates from another hospital district could be reduced by using an ambulance and a helicopter and how this affected their recovery.

**Methods:**

We prospectively gathered the time points of thrombectomy candidates referred to the Tampere University Hospital from the hospital district of Southern Ostrobothnia. Primary and secondary transports were included. In Hybrid transport, the helicopter emergency medical services (HEMS) unit flew from an airport near the CSC to meet the patient during transport and continued the transport to definitive care. Ground transport was chosen only when the weather prevented flying, or the HEMS crew was occupied in another emergency. We contacted the patients treated with mechanical thrombectomy 90 days after the intervention and rated their recovery with the modified Rankin Scale (mRS). Favourable recovery was considered mRS 0–2.

**Results:**

During the study, 72 patients were referred to the CSC, 71% of which were first diagnosed at the PSC. Hybrid transport (*n* = 34) decreased the median time from the start of transport from the PSC to the computed tomography (CT) at the CSC when compared to Ground (*n* = 17) transport (84 min, IQR 82–86 min vs. 109 min, IQR 104–116 min, *p* < 0.001). The transport times straight from the scene to CT at the CSC were equal: median 93 min (IQR 80–102 min) in the Hybrid group (*n* = 11) and 97 min (IQR 91–108 min) in the Ground group (*n* = 10, *p* = 0.28). The percentages of favourable recovery were 74% and 50% in the Hybrid and Ground transport groups (*p* = 0.38) from the PSC. Compared to Ground transportation from the scene, Hybrid transportation had less effect on the positive recovery percentages of 60% and 50% (*p* = 1.00), respectively.

**Conclusion:**

Adding a HEMS unit to transporting a thrombectomy candidate from a PSC to CSC decreases the transport time compared to ambulance use only. This study showed minimal difference in the recovery after thrombectomy between Hybrid and Ground transports.

**Supplementary Information:**

The online version contains supplementary material available at 10.1186/s12873-024-00931-0.

## Background

A large vessel occlusion (LVO) causing an ischemic stroke is not an uncommon finding [1] and is responsible for most stroke-related morbidity and mortality [2]. Intravenous thrombolysis (IVT) alone is ineffective in recanalizing a large artery of the brain [3], but when combined with mechanical thrombectomy, a near 100% reperfusion rate is achievable [4]. Recanalization of an occluded artery is more effective when less time has passed from the onset of stroke symptoms [5].

Much interest has been put into decreasing the in-hospital delays to the recanalization of stroke patients [6, 7]. There lies great potential for optimizing the prehospital timeline of patients suitable for mechanical thrombectomy distant to the comprehensive stroke centre (CSC) [8]. Guidelines recommend transporting stroke patients to the nearest primary stroke centre (PSC) except for patients with a contraindication to IVT [9]. The American Stroke Association’s policy statement update also suggests that stroke patients suspected of having an LVO could bypass a PSC if the travel time to the CSC increases not more than 15 min [10]. This is called the mothership strategy. Confirming the diagnosis of the LVO with computed tomography angiography (CTA) of the brain’s arteries in the PSC has some benefits. This so-called drip-and-ship strategy enables earlier administration of IVT and prevents the CSC from exceeding capacity. The RACECAT trial [11] showed that the time from the onset of symptoms to definitive treatment decreases by an hour with the mothership strategy. This study also showed that a 35-minute delay to the IVT did not reduce the odds of favourable recovery of patients with ischemic stroke.

The rural–urban inequality in access to undelayed treatment remains regardless of whatever transport strategy is used, and measures to decrease the transport time should be taken [12]. Leira et al. [13] called to action using helicopter emergency medical services (HEMS) to reduce the transport time to mechanical thrombectomy. Publications so far mostly compare the interfacility transfer times of patients referred for mechanical thrombectomy [14–16]. We found one study using HEMS in the mothership strategy [17] and one comparing the mothership and drip-and-ship strategies [18]. A common nominator for all these studies is that the patients await the helicopter’s arrival at the PSC or the location of the call-out of emergency medical services (EMS). Transports combining a ground ambulance and helicopter transfer have been described for thrombolysis candidates [19] but not for thrombectomy candidates from the scene or for interhospital transfers.

In this study, we present the time points for thrombectomy candidates referred to the Tampere University Hospital from a neighbouring hospital district. The patients were transported by ambulance only (Ground), or the transport began with an ambulance and continued by a HEMS unit (Hybrid). We also report the recovery rate of the patients treated with mechanical thrombectomy.

## Methods

### Study design

This prospective cohort study compared time points of different transport modalities from a rural hospital district to definitive care at the CSC. The times compared are clarified in a supplementary file.

### Study population

We prospectively included every thrombectomy candidate sent from the hospital district of Southern Ostrobothnia to Tampere University Hospital from June 1st, 2020, until October 10th, 2022. We stopped recruiting patients for this study as planned once a new HEMS base was established in the Southern Ostrobothnia region. We categorized the study population into four groups: (1) suspected LVO-patients whose transport started with an ambulance from the scene and continued via helicopter to the CSC (Mothership Hybrid), (2) suspected LVO-patients transported directly from the scene to the CSC by ambulance only (Mothership Ground), (3) confirmed LVO-patients transported first to the PSC, after which came transport to the CSC by ambulance and then continued via helicopter (Drip & Ship Hybrid), and (4) confirmed LVO-patients transported first to the PSC and then to the CSC by ambulance (Drip & Ship Ground).

### Setting

**Fig. 1:**
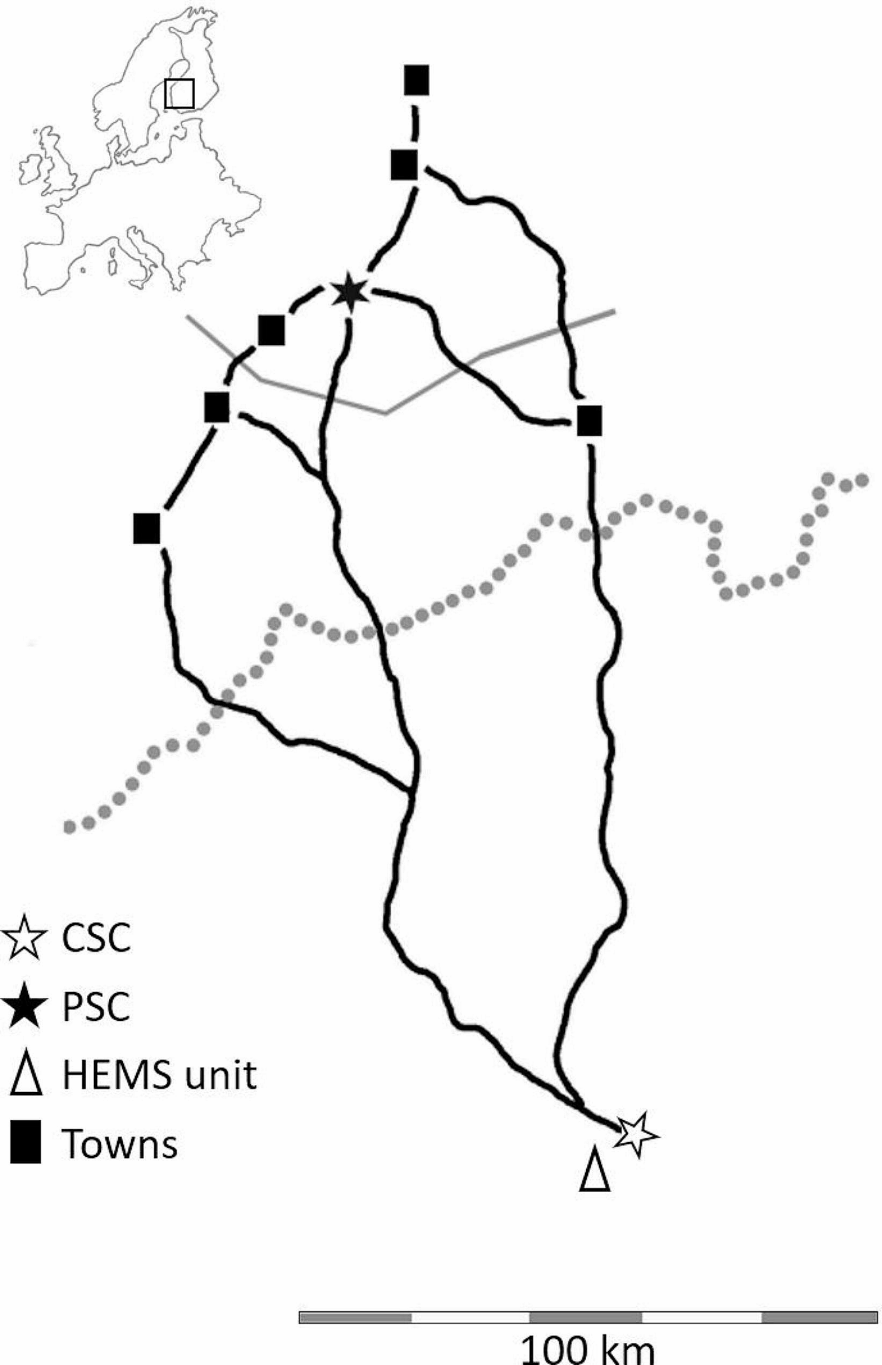
Map of the setting. The solid grey line depicts the border south of where the mothership strategy was executed. The dotted grey line is the border of the Southern Ostrobothnia hospital district. The black lines are the main roads from South Ostrobothnia to Tampere University Hospital. CSC comprehensive stroke centre, HEMS helicopter emergency medical services, PSC primary stroke centre

Tampere University Hospital is a CSC serving ca. one million people from the hospital districts of Kanta-Häme, Pirkanmaa, and Southern Ostrobothnia, Finland (Fig. 1). Circa 200 thrombectomies are done yearly at Tampere University Hospital.

The population of Southern Ostrobothnia’s hospital district is 190 000 in an area of 14 000 square kilometres. A considerable number of the people, 65 000, live in Seinäjoki. The surrounding municipalities are more rural, with a population density of fewer than 21 people per square kilometre. The emergency department (ED) of Seinäjoki Central Hospital is the only 24/7 ED with specialized care; it serves as a PSC in that hospital district. An on-call neurologist is available to make decisions about thrombolysis for acute stroke. The yearly number of stroke patients treated with thrombolysis is around 50. The distance between Tampere University Hospital and Seinäjoki Central Hospital is 150 km by air and 180 km via road.

The EMS in Southern Ostrobothnia’s hospital district encompasses 16 advanced life support level ambulances 24/7, 1 basic life support level (BLS) ambulance 24/7, 1 BLS ambulance for interhospital transport 8/5, and a community paramedic 12/7. There are 36 000 EMS call-outs yearly. The paramedics have been trained to recognize stroke using the Finnish Prehospital Stroke Scale (FPSS) [20]—a simple 5-item stroke assessment tool in which facial asymmetry, hemiparesis of a limb, speech difficulty, and visual disturbance each equals one point. If patients present with any of these symptoms without conjugate eye deviation away from the paretic side, they are considered candidates for thrombolysis. Conjugate eye deviation away from the side of the hemiparesis gives the patient an extra 4 points. Patients with 5 or more points in FPSS are candidates for thrombectomy. Ambulance crews encountering a patient with ≥ 5 FPSS points and a 20-minute drive with lights and sirens south from the Seinäjoki Central Hospital, i.e. towards the CSC, are advised to consult the on-call neurologist at the CSC. They discuss whether the patient could be considered for thrombectomy without confirming the diagnosis at the PSC. This is our study’s mothership strategy. EMS transport all other paramedic-suspected stroke patients to the PSC with a prehospital prenotification. When transporting a patient with ≥ 5 FPSS to the PSC, the paramedics are instructed to await the CTA’s result. The neurologist and the interventional radiologist at the CSC are consulted; thrombolysis is begun when applicable. The same paramedics continue the transport to CSC if the patient is suitable for mechanical thrombectomy. This is the drip-and-ship strategy. The CSC’s standard operating procedure considers any of the following arteries most likely accessible with mechanical thrombectomy devices when occluded alone or in combination: internal carotid artery, the first branch of the medial cerebral artery, the proximal part of the second branch of the medial cerebral artery, the proximal part of the anterior cerebral artery, basilar artery, vertebral artery, and the proximal part of the posterior cerebral artery. There was no restriction to the time from the onset of symptoms to arrival at the CSC. Penumbra was estimated with CT perfusion imaging.

FinnHEMS30 is a publicly funded, physician-led HEMS unit at the Tampere–Pirkkala Airport. The crew always consists of a pilot, a HEMS-paramedic, and a prehospital physician. Air operations are regulated by the European Union Aviation Safety Agency’s (EASA) guidelines and further specified by the Finnish Transport and Communication Agency (Traficom). They operate an EC135 helicopter with a cruising speed of 120kts. The landing zone requirement is 25 × 25 m in daylight and is doubled by night. The HEMS unit responds mainly to major trauma and out-of-hospital cardiac arrests but attends to other medical emergencies such as childbirth, intoxication, and severe bleeding without trauma. They attended 2000 call-outs and airlifted 30–40 patients per year before the implementation of this thrombectomy transport protocol. Previously, the HEMS unit was not dispatched to interhospital transfers. In June 2020, we dispatched FinnHEMS30 to expedite the transport of thrombectomy candidates to the CSC. Since the ability of the HEMS unit to transport the patient depends on weather conditions and not being active in some other HEMS call-outs, the ambulance crews were instructed to begin the transport without delay. The HEMS crew informed the ambulance on the radio if they could participate in the transport. The HEMS crew used a mobile application (Mapitare Easy Tracker, Mapitare Ltd, Finland) to track the ambulance’s real-time location and plan a suitable rendezvous. The patient handoff to the ED personnel occurred immediately at the heliport after the helicopter fully stopped.

### Outcome

Our primary aim was to compare the transport times between the Hybrid and Ground groups. We also gathered information about the recovery of the patients treated with thrombectomy. The CSC neurologists contacted all thrombectomy patients 90 days after the intervention and rated the patient’s recovery with the modified Rankin Scale (mRS) 0–6. For categorical comparisons, we considered mRS 0–2 “favourable” and the rest (mRS 3–6) as “poor” recovery.

### Statistical methods

We used the Microsoft Excel 2016 spreadsheet program (Microsoft Corporation, Redmond, USA) to gather prehospital timeline data from Codea web reporting (Codea Ltd, Porvoo, Finland) and the electronic patient records of the Pirkanmaa Hospital District. We used SPSS (IBM Corp., Armonk, NY, USA) statistical software version 26 to analyse the data. For categorical comparisons, we used a chi-square or Fisher’s exact test when applicable. For comparing continuous variables, we used the Mann–Whitney U test. The tests were two-sided, and *p* < 0.05 was considered significant.

The institutional review board of the Tampere University Hospital approved the study design (IRB number R20082R).

## Results

**Fig. 2:**
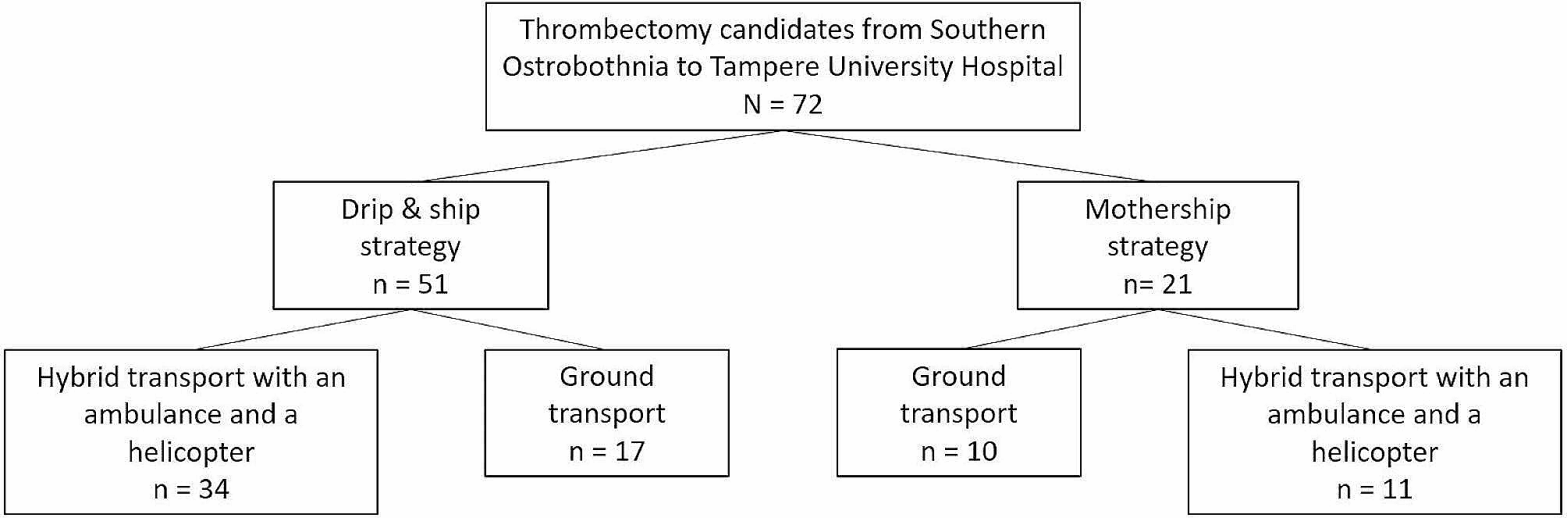
The flow diagram of the patients in the study with different transport methods

Seventy-two patients were referred to Tampere University Hospital from the Hospital District of Southern Ostrobothnia. Table 1 shows the patient demographics. The Hybrid transport method was used in 45 (63%) cases. The patient flow diagram is presented in Fig. 2. The most typical reason not to use a helicopter for transport was the weather conditions preventing the flight, which was the case 17 times (63%). One time (4%), there was no dispatch to the HEMS unit, despite the HEMS unit being available and the weather being good enough for flying.

**Table 1:** Patient characteristics

	Drip & ship *N* = 51		Mothership *N* = 21		p
	n / mean	% (SD)	n / mean	% (SD)	
Male	29	57	10	48	0.47
Age, years	69.7	(11.2)	75.3	(11.9)	1.00
Thrombolysis	31	61	2	9.5	**< 0.001**
Thrombectomy	33	65	9	43	0.12
Medical history					
Hypertension	34	67	12	57	0.44
Diabetes	13	25	3	14	0.37
Atrial fibrillation	12	24	6	29	0.65
Coronary disease	12	24	2	9.5	0.21
Previous stroke	5	10	3	14	1.00
Congestive heart failure	3	5.9	3	14	0.35
Renal failure	3	5.9	0	0	0.55
Dementia	0	0	2	9.5	0.08
Reason to choose ground transport					0.06
Weather condition	12	71	5	50	
HEMS priority	5	29	1	10	
No time gain	0	0	3	30	
Reason not treated with mechanical thrombectomy					0.09
No salvageable brain tissue	8	44	4	33	
Intracranial bleed	2	11	5	42	
Technical difficulties	3	17	3	25	
Clinical improvement	5	28	0	0	
Distance from scene to CSC, km	194.3	(27.2)	162.4	(17.5)	**< 0.001**
Straight line distance from scene to CSC, km	160.7	(24.8)	132.4	(16.4)	**< 0.001**

CSC comprehensive stroke centre, HEMS helicopter emergency medical services

Mothership strategy was used in 21 (29%) patients; the Hybrid transport was used for 11 of these patients. The mean distance from the scenes to the CSC in the mothership strategy groups was 162 km (SD 17 km) by road.

Ambulances using the drip-and-ship strategy spent a median of 12 min (IQR 8–16 min) on the scene, which was faster than the ambulances choosing the mothership strategy (median 23 min, IQR 17–28 min, *p* < 0.001). The call to recanalization time was longer with the drip-and-ship strategy than the mothership strategy (242 min, IQR 208–281 min vs. 170 min, IQR 148–203 min, *p* < 0.001). A considerable proportion of this difference is due to the door-in-door-out time (DIDO) time of a median of 46 min (IQR 35–62 min).

Table 2 shows the median times and interquartile ranges between the Hybrid and the Ground transports. The median Hybrid transportation time to the CSC scout was shorter only in the drip-and-ship strategy compared to the Ground transport (84 min, IQR 82–86 min vs. 109 min, IQR 104–116, *p* < 0.001).

**Table 2:** Median times (interquartile ranges) of patient flow with different transport methods

	Drip & Ship					Mothership				
	HEMS		GROUND		p	HEMS		GROUND		p
	*n* = 34		*n* = 17			*n* = 11		*n* = 10		
Onset to call	34	(2.5–79)	24	(4.0–54)	0.49	20	(1.5–209)	9	(3.0–62)	1.00
Call to scene	15	(7.0–18)	11	(89–16)	0.55	14	(7.0–19)	15	(11–19)	0.61
Onset to scene	52	(19–90)	43	(16–61)	0.73	41	(18–155)	31	(16–76)	**0.048**
On-scene time	12	(8.8–15)	12	(8.0–36)	0.65	23	(11–29)	24	(18–27)	0.61
Transport to the PSC	30	(13–49)	30	(11–36)	0.57					
Onset to the PSC	93	(68–166)	81	(55–107)	0.54					
Door to PSC scout	11	(8.0–11)	15	(10–18)	0.17					
Scout to ground transport time	34	(24–47)	32	(26–50)	0.98					
DIDO	45	(34–62)	50	(35–64)	0.77					
Ground transport to HEMS	41	(37–51)				53	(43–70)			
Patient loading on HEMS	8	(7.0–9.0)				7	(7.0–9.0)			
HEMS to the CSC	31	(25–31)				25	(22–31)			
Ground transport to the CSC			99	(98–105)				95	(82–102)	
Onset to the CSC	214	(180–338)	232	(214–263)	0.25	165	(128–253)	174	(132–195)	0.96
Arrival to CSC scout	11	(9.0–12)	7	(6.0–9.5)	**0.001**	11	(10–14)	7	(3.8–8.0)	**<0.001**
Transport to CSC Scout*	84	(82–86)	109	(104–116)	**<0.001**	93	(80–102)	97	(91–108)	0.28
Scout to recanalization*	44	(26–63)	41	(29–50)	0.63	41	(31–56)	55	(35–83)	0.41
Onset to recanalization	281	(241–436)	311	(284–537)	0.22	342	(222–696)	233	(187–257)	0.34

CSC comprehensive stroke centre, DIDO door-in-door-out time, HEMS helicopter emergency medical services, PSC primary stroke centre

Altogether, 42 (58%) patients were treated with mechanical thrombectomy. Two were not reached for mRS query. The patients reported a favourable recovery 90 days after the intervention as follows: Drip & Ship Hybrid group 17 (74%), Drip & Ship Ground group 4 (50%), Mothership Hybrid group 3 (60%), and Mothership Ground group 2 (50%). The differences in the percentages of favourable recovery were insignificant between the different transport methods in the drip and ship (*p* = 0.38) and mothership (*p* = 1.00) strategies. The percentage of patients with favourable recovery was also similar between the drip and ship and mothership strategies when the transport method was not considered (67% vs. 56%, *p* = 0.69).

## Discussion

In this study, we show that the Hybrid transport strategy of thrombectomy candidates taking advantage of a ground and a HEMS transport can decrease the transport time from a PSC to CSC. The time gain was 25 min when the straight-line distance between the two institutions was 150 km. Loading the patient onto the helicopter during transport from EMS call-outs nearer to the CSC facilities did not slow down the transport time to the CTA scout at the CSC when compared to the Ground transport group (93 and 97 min respectively, *p* = 0.28). However, helicopter utilization did not decrease the time from onset to recanalization meaning that every step in the stroke chain of survival should be refined further.

The difference in the on-scene times between the mothership and drip and ship strategies was interesting. With the drip and ship strategy, the paramedics could reach a median on-scene time of 12 min, but with the mothership strategy, the median on-scene time was extended to 23 min. Earlier, a standard for the on-scene time for EMS with stroke patients was set to 15 min [10], even though we know this goal is highly ambitious [21]. We do not have specific information about the location of the EMS call-outs in our study. We cannot rule out if the time difference is due to a more difficult evacuation of the patient or differing patient characteristics (e.g. patient’s weight or location on an upper floor), but it’s reasonable to believe that the need to consult the CSC neurologist from the scene is part of the delay [22]. The EMS tactic is more straightforward when the standard operating procedure of transporting a stroke patient has only one destination: the PSC. In a small urban cohort, fire engine support at the scene of a stroke patient did not decrease the on-scene time [23]. This could differ in a rural environment if the LVO is already recognized during the emergency call [24] and a first responder unit is dispatched with the EMS. In this case, the ALS paramedic could consult the neurologist while the first responder unit executes evacuating the patient to the ambulance.

The additional delay at the scene was insignificant when comparing the time to definitive treatment between the mothership and drip and ship strategies. The patients reached the CSC considerably faster when the PSC was bypassed. This was also the case in, for example, the RACECAT trial [11]. Our PSC’s DIDO performance was excellent. McTaggart et al. [25] report a considerable decrease in their institution’s DIDO from a median of 104 to 64 min after an intensive implementation of a new protocol. In Hädrich et al.’s report [16], the median time from imaging at the PSC to transfer request was around 50 min; the transport’s departure after the request took an extra half hour. Even when the drip and ship strategy is used, the paramedics should be trained to suspect LVO when transporting a stroke patient to the PSC. In our protocol, the same ambulance continues with the stroke patient to the CSC. We suggest there is no need for a retrieval team or a separate transfer request in these time-critical situations.

A critical decision point in the drip and ship strategy is the EMS personnel’s prenotification concerning a thrombectomy candidate. Contraindications to IVT should be discussed, and bypass of the PSC considered when feasible [9]. Twenty patients in our drip and ship strategy did not receive IVT at the PSC. Their stop included only confirmation of the LVO and consultation with the CSC. With the mothership strategy, 24% of patients were diagnosed with haemorrhagic stroke, and no patients with a stroke mimic in favour of FPSS. Unfortunately, one-fourth of the patients with LVO in the mothership strategy arrived too late for thrombectomy.

We defined the transport time beginning from the departure to the CT scout because the helicopter landing site at the CSC’s roof is further away from the CT at the emergency department than the ambulance garage. Then again, the neurologists at the CSC met the patient at the top of the hospital, and the patient status had already been checked in the elevator down to the CT room. A discrepancy exists in how earlier studies report the time gain of the HEMS unit. Hence, comparing our results to previous studies is difficult. Regenhardt et al. [14] report the time from the patient’s last known normal to CSC arrival; in their report, the HEMS group travels a longer distance than the ground group. Hädrich et al. [16] report the time from the transfer request to the CSC door and state that the waiting time for the helicopter to arrive at the PSC is overthrown only when the distance between the PSC and CSC is over 71 km. Almallouhi [15] and Kunte [18] report the transport time to arrival at the CSC. Imahori et al. [17] report the time from the emergency call to admission at the CSC in the mothership strategy but fail to report how the HEMS unit is dispatched to the scene.

A common finding is that the advantage of the HEMS unit depends on the distance from the patient to the CSC. Local circumstances, such as traffic and driving conditions, define the distance when the HEMS should be used. Imahori et al. [17] interpolate that the time from the emergency call to the arrival at the CSC is already faster with HEMS when the distance from the CSC is beyond 10 km. The time spent loading the patient onto the helicopter in the field and off the helicopter at the CSC must be considered. In our population, the last place to load the patient onto the helicopter is roughly a 60-minute drive with lights and sirens away from our CSC– a sawmill’s parking place 100 km by road and 80 km by air from the CSC. Most of the patients in the drip and ship strategy’s Hybrid group were loaded onto the helicopter about 40 km further on a truck parking place beside a gas station. These distances could show a 25-minute decrease in travel time to the CSC scout in the drip and ship strategy and no time gain in the mothership strategy. The distances in our study are greater than in previous reports. For example, in Hädrich et al.’s [16] report, one-fifth of patients travel over 100 km; in Almallouhi et al.’s [15] report, the furthest quartile is transferred more than 150 km. The helicopter’s early dispatch plays an important role when the travel time is reduced.

The patient’s recovery after an LVO is mainly determined from the onset of symptoms to recanalization [5]. This timeline can be roughly divided into three stages of delays: the delay from the onset of symptoms to the emergency call, prehospital delay (from the call to the arrival at definitive care), and in-hospital delay at the CSC. We anticipated that our time gain with the hybrid transport from the onset of symptoms to recanalization and our population size would not add up to any clinical benefit. Yet we are satisfied to report the overall good performance and a favourable recovery trend of our thrombectomy candidate transport protocol from another hospital district. Even from distant locations, 58% of our patients received the treatment, with 62% having a favourable recovery. In their international meta-analysis with hundreds of patients, Saver et al. [5] report an odds ratio of 0.81 for mRS 0–1 for every hour of delay from the onset of symptoms to recanalization with mechanical thrombectomy. If we wanted to achieve an hour reduction in time to recanalization, we would have to bypass the PSC using Hybrid transportation. The RACECAT trial [11] had a 56-minute decrease from the onset of symptoms to the groin puncture at the CSC when the PSC was bypassed, but the outcome reporting is limited to all patients diagnosed with an ischemic stroke and not to those treated with thrombectomy. Hubert et al. [26] reported almost a 100-minute decrease in the delay from the onset of symptoms to reperfusion when utilizing a flying intervention team; however, even this decrease in the delay was insufficient to show a decrease in mRS in a study population of 117 patients. Their study design required a considerable investment in an extra helicopter and the possibility for the interventionalist team to depart their hospital.

The small number of patients and single-centre design limits our study. The number of patients in our study was insufficient to show any clinical benefit of a 25-minute decrease in transport time. Yet the decrease accomplished encourages us to further develop the transport protocol of thrombectomy candidates now that there is a new HEMS base nearer to the PSC at Seinäjoki. We also extended the same hybrid transport of thrombectomy candidates from other hospital districts that are even further from our CSC. It was reasonable to believe that this small pilot study is feasible but expanding it to other hospital districts might not be as straightforward. Silliman et al. [19] highlight the need for the repetitive training this protocol implementation requires.

The non-randomized design of this report can also be criticized. We aimed at primarily utilizing the HEMS unit, and Ground transport was chosen only when the helicopter was not available. The regulations that restrict flying in certain weather conditions do not necessarily mean that driving with lights and sirens would be slower than usually. For example, only the probability of the ceiling coming down to 300ft in the day and to 900ft in the night would hinder the flight with no impact to the ambulance drive time. Local freezing fog at the airport prevents even takeoff with instrument flight rules but still the ambulance could have excellent driving conditions. Therefore, we regard this reporting is feasible. We did consider a before-after design with historical transport data for comparison, but this design does have its downsides as well.

## Conclusion

Our study shows that it is possible to decrease the transport time of a thrombectomy candidate from another hospital district to the CSC when the transfer involves ground and air transport.

### Electronic supplementary material

Below is the link to the electronic supplementary material.

Supplementary Material 1

## Data Availability

Depersonalized data can be made available from corresponding author upon a reasonable request.

## Abbreviations

ALS: Advanced life support
BLS: Basic life support
CTA: Computed tomography angiography
CSC: Comprehensive stroke centre
DIDO: Door-in-door-out
ED: Emergency department
EMS: Emergency medical services
FPSS: Finnish prehospital stroke scale
HEMS: Helicopter emergency medical services
IVT: Intravenous thrombolysis
IQR: Interquartile range
LVO: Large vessel occlusion
mRS: Modified Rankin Scale
PSC: Primary stroke centre
