# Supplementary material for: A hybrid strategy using an ambulance and a helicopter to convey thrombectomy candidates to definite care: a prospective observational study
Source: BMC Emerg Med. 2024 Jan 25;24:17. doi: 10.1186/s12873-024-00931-0 (PMC10809465; doi:10.1186/s12873-024-00931-0)
Supplement: Supplementary file 1 — Supplementary Material 1 [file 12873_2024_931_MOESM1_ESM.docx]

Table. Definitions of time points in the study

| **Time point** | **Definition** |
| --- | --- |
| Onset to call | Time from the patient’s last known normal until the beginning of the emergency call |
| Call to scene | Time from the beginning of the emergency call to the first ambulance’s arrival at the scene |
| On-scene time | Time from the ambulance’s arrival to the start of the transport |
| Transport to PSC | Time from the start of transport to the arrival at the PSC |
| Time to PSC scout | Time from the ambulance’s arrival at the PSC to the time of the head CT scout at the PSC |
| Scout to ground transport time | Time from the CT scout to starting the ambulance transport to the CSC |
| DIDO time | Time from the ambulance’s arrival at the PSC to the ambulance’s departure to CSC |
| Ground transport to the CSC | Time from the start of transport from the scene or from the PSC to the arrival at the CSC |
| Ground transport to HEMS | Time from the start of transport from the scene or from the PSC to rendezvous with the HEMS unit |
| Patient loading on HEMS | Time from the ambulance’s arrival at the rendezvous to the helicopter’s departure to the CSC |
| HEMS to the CSC | Time from the start of the air transport to the time of the CT scout at the CSC |
| Time to CSC scout | Time from the arrival at the CSC to the time of the head CT scout at the CSC |
| Scout to recanalization | Time from the CT scout at the CSC to the time of “post angiogram” in the intervention suite |
| Onset to recanalization | Time from the onset of symptoms to the time of “post angiogram” in the intervention suite |

CSC comprehensive stroke centre, CT computed tomography, DIDO Door-in-Door-out, HEMS helicopter emergency medical services, PSC primary stroke centre
